# FGF14 Functions as a Tumor Suppressor through Inhibiting PI3K/AKT/mTOR Pathway in Colorectal Cancer

**DOI:** 10.7150/jca.36316

**Published:** 2020-01-01

**Authors:** Tianhong Su, Linlin Huang, Ning Zhang, Sui Peng, Xiaoxing Li, Guangyan Wei, Ertao Zhai, Zhirong Zeng, Lixia Xu

**Affiliations:** 1 Department of Liver Surgery, The First Affiliated Hospital, Sun Yat-sen University, Guangzhou, Guangdong, China;; 2 Department of Gastroenterology and Hepatology, The First Affiliated Hospital, Sun Yat-sen University, Guangzhou, Guangdong, China;; 3 Department of Gastroenterology and Hepatology, Guangdong Provincial People's Hospital/Guangdong Academy of Medical Sciences, Guangzhou, Guangdong, China;; 4 Clinical Trials Unit, The First Affiliated Hospital, Sun Yat-sen University, Guangzhou, Guangdong, China;; 5 Precision Medicine Institute, The First Affiliated Hospital, Sun Yat-sen University, Guangzhou, Guangdong, China.; 6 Department of Gastrointestinal Surgery, The First Affiliated Hospital, Sun Yat-sen University, Guangzhou, Guangdong, China;; 7 Department of Oncology, The First Affiliated Hospital, Sun Yat-sen University, Guangdong, China

**Keywords:** FGF14, DNA methylation, tumor suppressor, apoptosis, colorectal cancer

## Abstract

We identified that Fibroblast Growth Factor 14 (FGF14) was preferentially methylated in colorectal cancer (CRC). In this study, we aimed to investigate the epigenetic regulation, biological function and molecular mechanism of FGF14 in CRC. The expression of FGF14 in CRC cell lines, normal human colon epithelial cell line, CRC tissues and paired adjacent normal tissues was detected by PCR and Western blot. The biological function of FGF14 in CRC was interrogated by cell viability assay, colony formation, flow cytometry, cell invasion and migration assay, as well as *in vivo* study. We found FGF14 was downregulated or silenced in all (10/10) CRC cell lines, while it was expressed in normal colonic tissues and normal human colon epithelial cell line. The expression of FGF14 was lower in primary CRCs as compared to their adjacent normal tissues. Significant higher methylation of FGF14 was observed in CRCs than that in normal tissues based on the data from TCGA database. The loss of FGF14 gene expression was restored by treatment with DNA methyltransferase inhibitor 5-Aza. Re-expression of FGF14 in CRC cell lines inhibited cell viability and colony formation, and induced cell apoptosis. FGF14 induced mitochondrial apoptosis and inhibited PI3K/AKT/mTOR pathway. In xenograft mouse model, overexpression of FGF14 significantly reduced tumor growth (*P*<0.001). In conclusion, FGF14 is a novel tumor suppressor, which suppresses cell proliferation and induces cell apoptosis* via* mediating PI3K/AKT/mTOR pathway.

## Introduction

Colorectal cancer (CRC) is one of the most common malignancies worldwide 1. In Asian countries, the incidence of CRC has been rising rapidly 2. The pathogenic mechanism underlying CRC development appears to be complex and heterogeneous, which is a multistep process with the accumulation of genetic and epigenetic alterations 3, 4. Emerging evidence indicates that promoter methylation of genes associated with gene silencing, plays an important role in the development and progression of CRC 5-8. The identification of novel tumour-suppressive genes targeted by promoter methylation may help to reveal tumour-suppressive pathways in colorectal carcinogenesis and find alternative approaches for diagnostic and therapeutic evaluation.

Through a genome-wide screening, we identified that Fibroblast growth factor 14 (*FGF14*) was frequently silenced by promoter methylation in CRC. This gene is located on chromosome 13q33 and belongs to the fibroblast growth factor (FGF) family 9, 10. Through de-novo function prediction approach, FGF14 is found to be localized to the nucleus 11. As an intracellular protein controlling neuronal excitability and synaptic transmission, FGF14 was reported to be correlated with neurologic and psychiatric disorders 12, 13. Until now, the role of FGF14 in colorectal carcinogenesis remains unknown. In this study, we studied the expression of *FGF14* in human CRC and its promoter methylation to determine whether epigenetic inactivation of *FGF14* exists in CRC. We further investigated its biological function in CRC through *in vitro* and *in vivo* experiments. Finally, the molecular mechanism of its biological function in colorectal tumorigenicity was evaluated.

## Materials and Methods

### Primary tumor and normal tissue samples

Ethical approval for human subjects was obtained from the Institutional Review Board of the First Affiliated Hospital, Sun Yat-Sen University (FAHSYSU), and written consent was obtained from each patient. Paired specimens from primary colorectal cancer and adjacent nontumor sites were obtained from 13 CRC patients at the time of operation.

### Tumor cell lines

Ten colorectal cancer cell lines (CaCO2, CL4, DLD-1, HCT116, HT29, LOVO, LS180, SW480, SW620 and SW1116), one normal human colon epithelial cell line (NCM460) and mouse embryonic fibroblasts (MEF) cell line were used in this study. All the cell lines applied were acquired from the Type Culture Collection of the Chinese Academy of Sciences, Shanghai, China, which were all proved to be free from mycoplasma contamination and were authenticated by short tandem repeat (STR) analysis. Cells were cultured in RPMI 1640 medium (Gibco BRL, Rockville, MD, USA) supplemented with 10% fetal bovine serum (Gibco BRL).

### RNA extraction, semi-quantitative RT-PCR and real-time PCR analyses

Total RNA was extracted from cell pellets and tissues using Quizol reagent (Qiagen, Valencia, CA). Semi-quantitative RT-PCR was performed using the Go-Taq DNA polymerase (Promega, Madison, WI) with the housekeeping gene GAPDH as an internal control. Real-time PCR was performed using SYBR Green master mixture on HT7900 system according to the manufactures' instructions (Applied Biosystems) with GAPDH as an internal control. Primer sequences were listed in Table S1.

### DNA extraction, Bisulfite treatment of DNA, Methylation-Specific PCR (MSP)

Genomic DNA was extracted from the cell pellets and tissues using QIAamp DNA Mini kit (Qiagen, Hilden, Germany). DNA was chemically modified with sodium metabisulphite as previously described 14. The bisulfite-modified DNA was amplified by using primer pairs that specifically amplify either methylated or unmethylated sequences of the* FGF14* genes (Table S1). MSP was performed for 40 cycles using the Taq-Gold polymerase (Applied Biosystems). Primer sequences were listed in Table S1.

### Western Blot analysis

Total protein was extracted from stably transfected cells and protein concentration was measured by the DC protein assay method of Bradford (Bio-Rad, Hercules, CA). 30 micrograms of protein from each sample were separated Sodium dodecyl sulfate-polyacrylamide gel electrophoresis (SDS-PAGE) and transferred onto nitrocellulose membranes (GE Healthcare, Piscataway, NJ). The dilution of primary antibodies was according to the company's recommendation. Antibodies information were listed in Table S2. Proteins were visualized using ECL Plus Western blotting Detection Reagents (RPN2132, GE Healthcare, Piscataway, NJ).

### 5-Aza-2'-deoxycytidine (5-Aza) treatment

Colorectal cancer cells were seeded at a density of 1×10^6^ cells/mL. After overnight culture, cells were treated with 2 µM of the DNA methyltransferase inhibitor 5-aza-2'-deoxycytidine (Aza) (Sigma, St. Louis, MO) for 96 hours. After treatment, cells were harvested for DNA and RNA extractions.

### Construction of FGF14 expression plasmid

Complementary DNA corresponding to the full-length *FGF14* was obtained by RT-PCR amplification with primers specific to *FGF14*. The PCR aliquots were subcloned into mammalian expression vector LV003 containing a GFP-tag (Forevergen, Guangzhou, China). The LV003 plasmid was used as the vector control in transfection experiments. *FGF14* expression construct was verified by genomic sequencing.

### Cell viability assay

Cell viability was determined by cell counting Kit-8 (CCK-8) assay (Dongjido, Japan). Briefly, the cells were stably transfected with expression plasmids-LV003-*FGF14* or the empty vector LV003 in a 96-well plate for 1, 2, 3 days, respectively. 10 µl of reaction solution and 90 ul RPMI 1640 medium were added to cells. The mixture was incubated at 37°C for 1 h. The optical density was measured at a wavelength of 450 nm.

### Colony formation assay

DLD1 and HCT116 cells were transfected with expression plasmids LV003-*FGF14* or the empty vector LV003 using lipofectamine 2000 (Invitrogen). After 48 h of transfection, cells were replated and selected with G418 at 0.5 mg/mL for 10-14 days. Colonies (≥ 50 cells/colony) were counted after fixed with 70% ethanol and stained with crystal violet solution.

### Migration and Matrigel invasion assays

For migration assay, cells were seeded into the upper chamber of a Transwell insert (pore size, 8 μm; Corning Falcon) and then placed into the transwell containing medium with 10% FBS in the lower chamber. For invasion assay, cells were seeded in a Matrigel-coated chamber (Becton Dickinson, Waltham, MA, USA). After 48h, cells that remained in the lower surface of the insert were stained with crystal violet.

### Flow cytometry

Apoptosis was assessed by flow cytometry after staining with Annexin V (FITC-conjugated; BD, Belgium) and PI (BD). The stably transfected CRC cells with LV003-*FGF14* vector or LV003 empty vector were stained and incubated for 20 min at room temperature in darkness. The results were analyzed using an Epics Profile II flow cytometer (Beckman Coulter, Fullerton, CA) and Multicycle software (Phoenix Flow Systems, San Diego, CA).

### *In vivo* tumorigenicity

HCT116 cells (1×10^7^ cells in 0.1 mL PBS) transfected with *FGF14* or LV003 were injected subcutaneously into the dorsal left flank of 4-week-old male Balb/c nude mice (5/group). Tumor diameter was measured every 2-3 days until 4 weeks. Tumor volume (mm^3^) was estimated by measuring the longest and shortest diameter of the tumor. All animal use was approved by the institutional care and animal use committee of FAHSYSU.

### Statistical Analysis

The SPSS version 18.0 (SPSS Inc, Chicago, IL) was used for analysis of the data. The results were expressed as mean ± standard deviation (SD). The difference in tumor growth rate between the 2 groups of nude mice was determined by repeated-measures analysis of variance. Value of *P* < 0.05 was taken as statistical significance.

## Results

### FGF14 was downregulated and methylated in CRC

**Figure 1:**
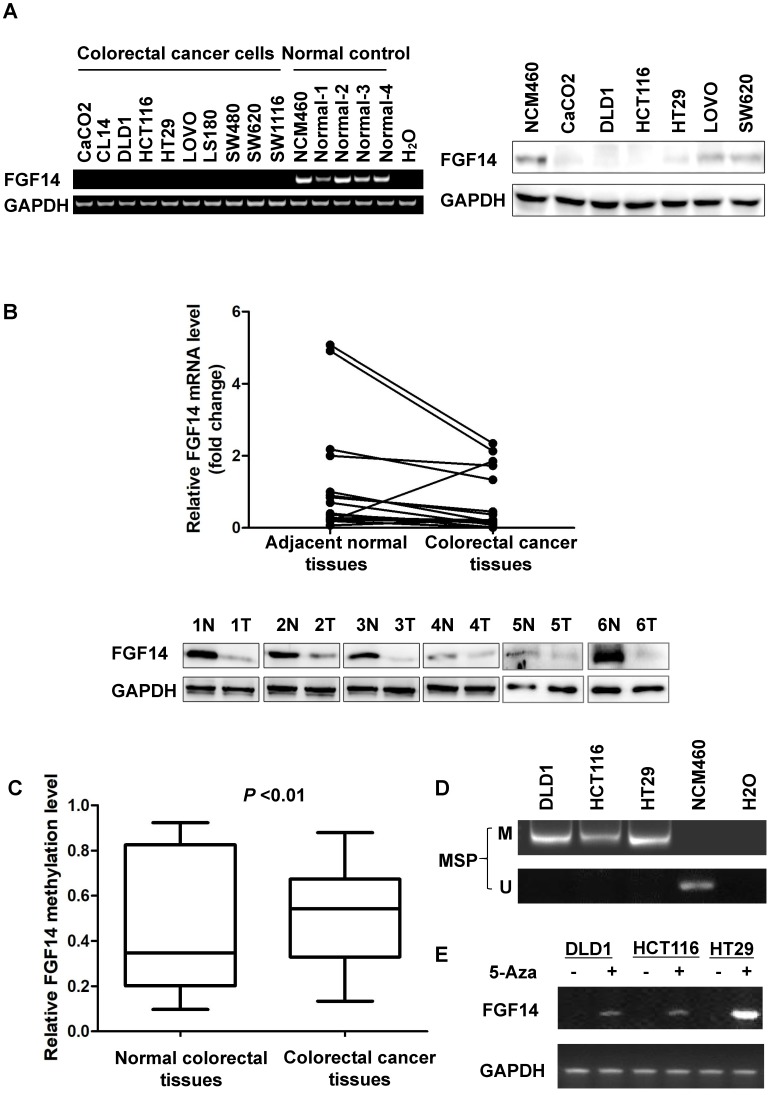
** Promoter methylation of FGF14 led to the downregulation of FGF14 in colorectal cancer (CRC) tissues and cell lines.** (A) Expression of FGF14 mRNA (left panel) and protein (right panel) was significantly reduced in CRC cell lines compared with normal control. (B) Expression of FGF14 mRNA (upper panel) and protein (lower panel) were significantly downregulated in tumor tissues compared with matched adjacent normal control in 13 CRC cases, respectively. (C, D) Significant higher methylation of FGF14 was observed in CRCs than that in normal tissues based on the data from TCGA database (N=45) and in CRC cell lines in comparison with normal human colon epithelial cell line NCM460. (E) Treatment of DNA methyltransferase inhibitor 5-Aza restored the expression of FGF14 in CRC cell lines. **p<0.01.

We found FGF14 was downregulated or silenced in all (10/10) CRC cell lines, while it was expressed in normal colonic cell line NCM460 and tissues through RT-PCR, which was validated by Western blot in six CRC cell lines including CaCO2, DLD1, HCT116, HT29, LOVO and SW620 and in normal colon epithelial cell line NCM460 (Fig. 1A). The expression of FGF14 was lower in primary CRCs as compared to their adjacent normal tissues in 13 CRC cases through Real-time PCR and Western blot (Fig. 1B).

We next explored FGF14 methylation status in CRC tissues and normal tissues from TCGA database. Significant higher methylation of FGF14 was observed in CRCs than that in normal tissues (*p*<0.01) (Fig. 1C). Consistently, full methylation was also found in CRC cell lines (Fig. 1D, Fig. S1). To determine whether the promoter methylation mediates the silencing of FGF14, three CRC cell lines (DLD1, HCT116 and HT29) were treated with the DNA methyltransferase inhibitor 5-Aza. Expression of FGF14 was restored in these three CRC cell lines (Fig. 1E). Therefore, our results suggested that the transcriptional silence of FGF14 was regulated by DNA methylation.

### FGF14 suppressed cell growth and promoted cell apoptosis of CRC

**Figure 2:**
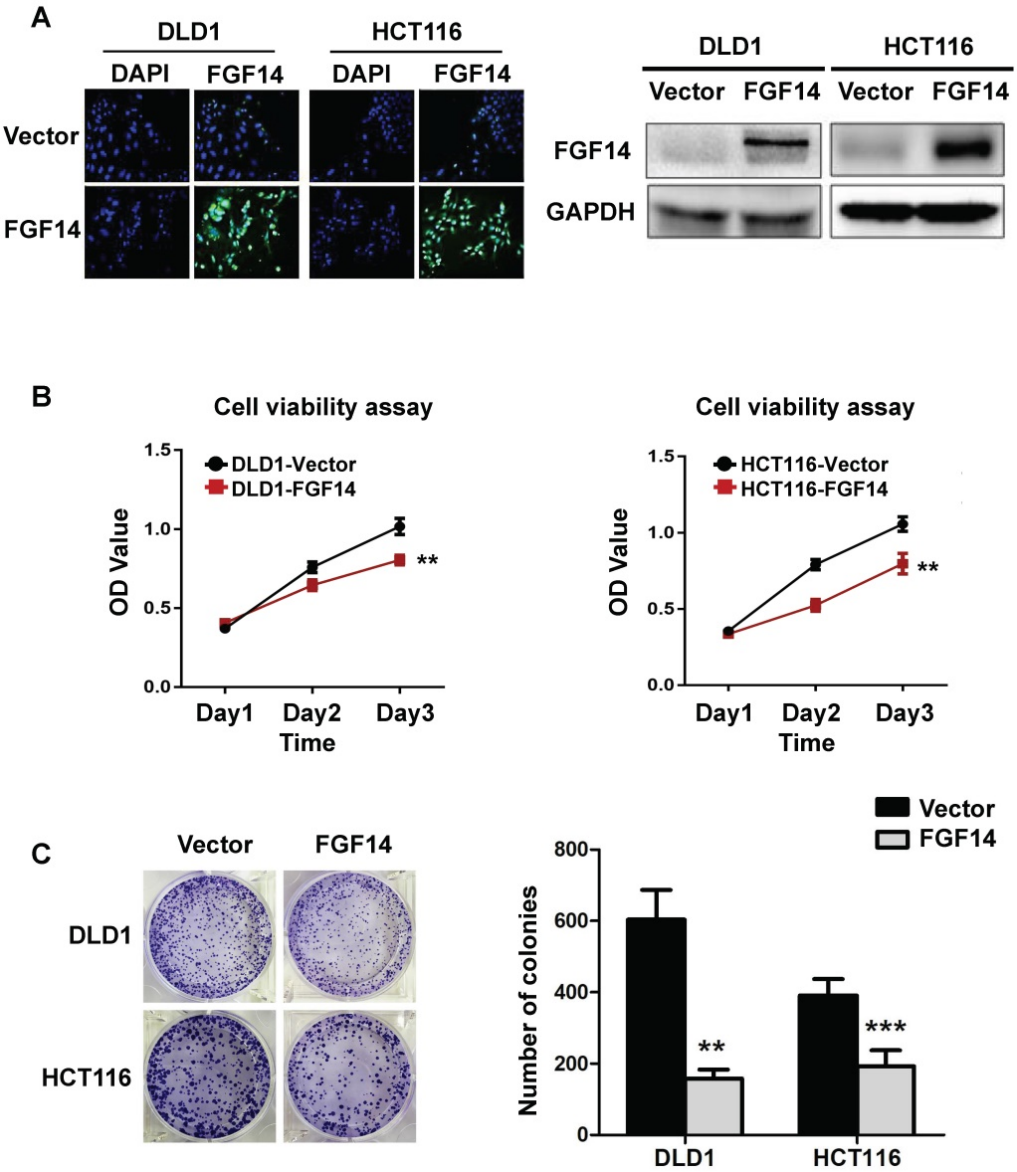
** FGF14 inhibited cell growth of CRC.** (A) Representative fluorescence image of FGF14 overexpression showed that transfection efficiency was high in DLD1 and HCT116 (Left panel). Ectopic expression effects of FGF14 were validated by Western blot (right panel). (B) The overexpression of FGF14 significantly suppressed cell viability in DLD1 and HCT116 compared with control, as determined by CCK8 assay. (C) The ability of colony formation in FGF14-transfected DLD1 and HCT116 cells significantly decreased as compared to empty vector-transfected control. *p<0.05, **p<0.01, ***p<0.001.

The high frequency of FGF14 silencing in both CRC cell lines and tissues suggests a tumor-suppressive role of FGF14 in CRC tumorigenesis. To testify that, we constructed an overexpression plasmid of FGF14 and performed transfection upon CRC cell lines (DLD1 and HCT116) through Lipofectamin 2000, with Vector-infected cells as control. The transfection efficiency and subsequent ectopic expression effects of FGF14 were validated by immunofluorescence and Western blot, respectively (Fig. 2A). The overexpression of FGF14 significantly attenuated cell viability as compared with control cells by CCK8 assay (Fig. 2B). Moreover, the capability of colony formation in FGF14-transfected cells decreased drastically in comparison with empty vector-transfected DLD1 and HCT116 (Fig. 2C). The abovementioned data indicated that FGF14 was negatively correlated with CRC cell growth. However, depletion of FGF14 influenced little on cell proliferation or colony formation capability in MEF cells, indicating a versatile role of FGF14 in normal and malignant cells (Fig. S2).

### FGF14 induced mitochondrial apoptosis and inhibited PI3K/Akt/mTOR signaling pathways

**Figure 3:**
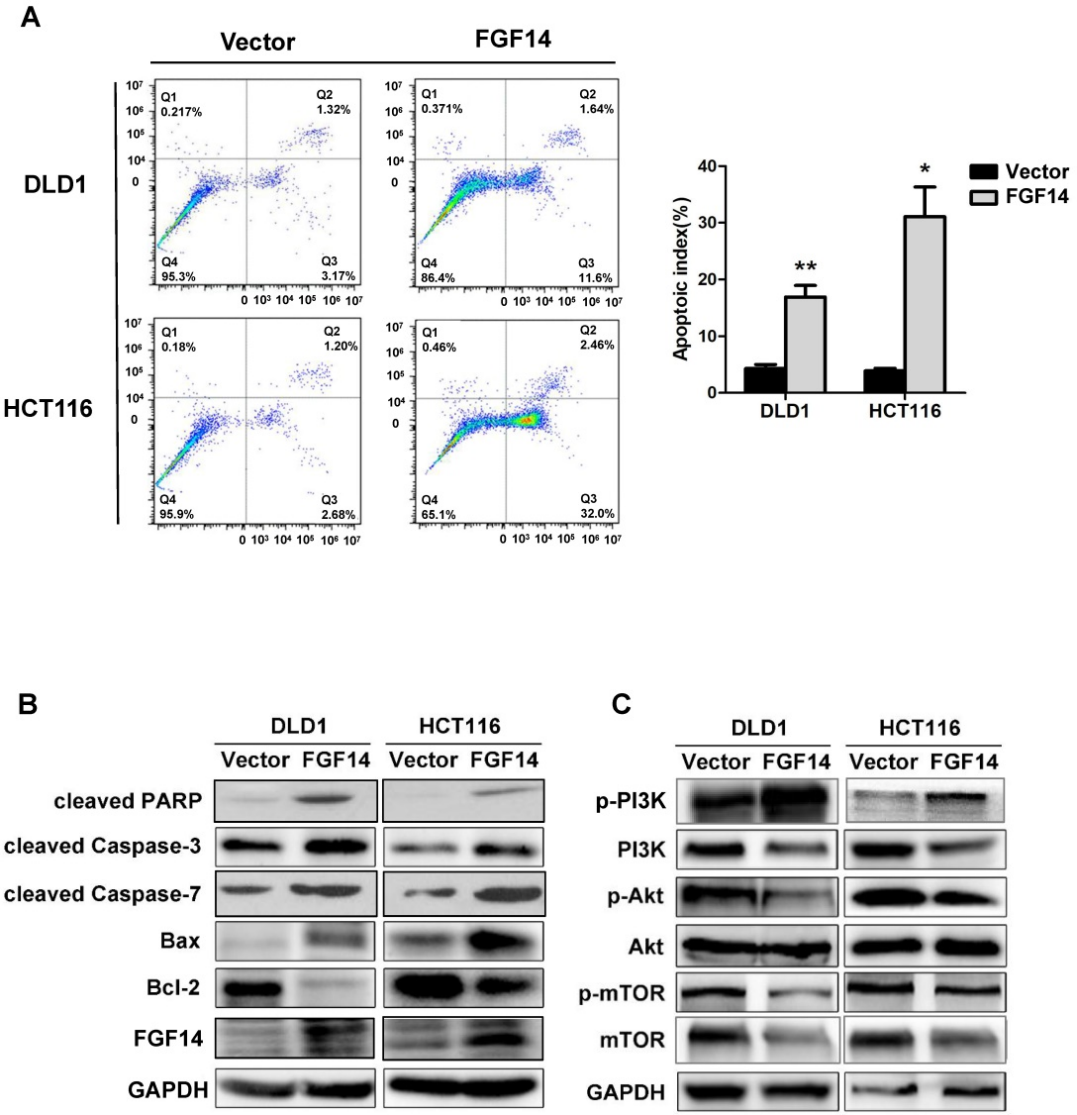
** FGF14 induced mitochondrial apoptosis through mediating PI3K/AKT/mTOR signaling pathways.** (A) Ectopic expression of FGF14 significantly increased CRC cell apoptosis. (B). Pro-apoptotic and anti-apoptotic markers were evaluated by Western blot. (C) PI3K, phosphorylated AKT and phosphorylated mTOR were downregulated with ectopic expression of FGF14 in both CRC cell lines. *p<0.05, **p<0.01.

Next, we further evaluated whether or not apoptosis is involved in the compromised cell growth by FGF14. Flow cytometry with annexin V and PI double staining was performed to evaluate the effect of FGF14 on apoptosis. Apoptosis in CRC cells were greatly increased by FGF14 ectopic expression, demonstrated by a significant increase in the percentage of annexin-V-positive cells (Fig. 3A). These results indicate that FGF14 promoted cell apoptosis of CRC. However, neither migration ability nor invasion ability of CRC cells was affected by the ectopic expression of FGF14, as shown by migration and invasion assays (Fig. S3A and S3B).

Suppression of cell growth in tumor cells is usually associated with concomitant activation of cell death pathways. To determine the manner by which FGF14 induces CRC cell apoptosis, we performed Western blot against typical mitochondria apoptotic markers. We found that specific cleavage of poly (ADP-ribose) polymerase (PARP), cleaved-caspase-3, cleaved-caspase-7, as well as Bax were significantly induced by FGF14 overexpression, while the anti-apoptotic factor Bcl-2 level was decreased (Fig. 3B). Taken together, this data suggested that FGF14 induced cell apoptosis *via* the mitochondria pathways in CRC.

PI3K/AKT pathway is a well-established cell survival pathway that was found associated with anti-apoptotic cell growth. We further investigated whether FGF14 induced mitochondrial apoptosis through interacting with PI3K/AKT pathway. Western blot analysis showed that the protein expression of PI3K decreased in CRC cells with FGF14 overexpression (Fig. 3C). Concomitantly, the expression of phosphorylation of AKT was downregulated (Fig. 3C). We further explored the impact of FGF14 on mTOR, an important downstream executor of AKT. FGF14 overexpression led to a marked decrease in phosphorylated mTOR (Fig. 3C). These data together implied that FGF14 induced mitochondrial apoptosis through inactivating PI3K/AKT/mTOR signaling pathway in CRC.

### FGF14 inhibited tumorigenicity in nude mice

**Figure 4:**
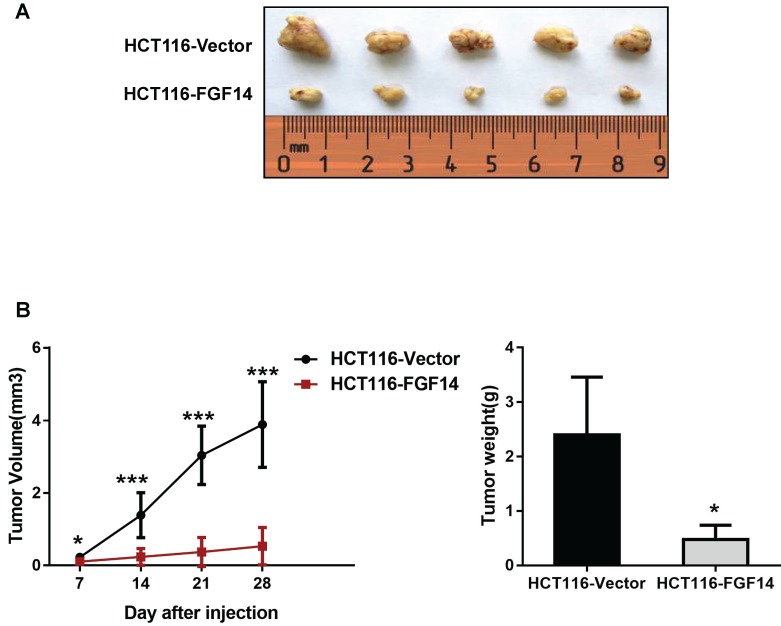
** FGF14 inhibited tumorigenicity *in vivo*** (A) The nude mice injected with HCT116-FGF14 cells showed a significantly slower tumor growth rate than those in the control group. (B) Mice injected with HCT116-FGF14 cells presented with smaller and lighter tumors in comparison with those in the control group. *p<0.05, **p<0.01, ***p<0.001.

To further explore the tumorigenic ability of FGF14 in *vivo*, empty vector-transfected and FGF14-transfected HCT116 cells were inoculated into the right flanks of nude mice, respectively. The tumor growth rates in the nude mice injected with HCT116-FGF14 cells were significantly slower than those in the control group (Fig. 4A).

Twenty-eight days after injection, the mice were sacrificed, and the xenografts were excised to assess the average tumor volume and weight in each group. We found that mice injected with HCT116-FGF14 cells possessed smaller (0.53 ± 0.52 mm^3^ *vs* 3.89 ± 1.18 mm^3^) and lighter (0.48 ± 0.26 g *vs* 2.4 ± 1.06 g) xenografts in comparison with those in the control group (Fig. 4B). The results from the* in vivo* model provided further evidence of the tumor-suppressive role of FGF14.

## Discussion

In this study, we first illustrated that FGF14 was downregulated in CRC tumor tissues and cell lines, which is probably attributed to promoter methylation of FGF14. Expression of FGF14 was successfully restored after the demethylation treatment by DNA methyltransferase inhibitor 5-Aza, suggesting that promoter methylation of FGF14 plays an important role in the transcriptional silence of FGF14 in CRC. We further demonstrated that FGF14 might act as a tumor suppressor which inhibited cell growth and induced cell apoptosis of CRC through mediating PI3K/AKT/mTOR signaling pathway.

FGF14 is a member of FGFs family, which are mainly found to regulate both neural and mesodermal cell fates during the development of embryo 15. Recent studies have extended the physiological roles of FGF signaling on regulating angiogenesis and wound healing in the adult organism. Receptors of FGFs are found expressed on various cell types and influence principle cell behaviors from proliferation, differentiation to survival, making FGF signaling susceptible to be subverted by malignant cells 16. Limited studies have explored the role of FGFs on tumorigenesis process, especially for FGF14. FGF14 is a neural system tropic factor and exerts its function mainly in the nervous compartment, related with neurologic and psychiatric disorders 12, 13, 17. In this study, we firstly explored the epigenetic regulation, biological function and molecular mechanism of FGF14 in CRC.

Transcriptional silence upon the promoter methylation of tumor suppressor genes has been reported to be involved in the tumorigenesis and progression of CRC 18-21. We observed low or silenced expression of FGF14 in both CRC tumor tissues and cell lines, accompanying promoter methylation of FGF14, suggesting that promoter methylation might be the predominant mechanism for the inactivation of FGF14 in CRC.

We further found FGF14 inhibited cell growth and promoted cell apoptosis of CRC through* in vivo* and* in vitro* experiments. These results indicate for the first time that FGF14 functions as a tumor suppressor in CRC. The tumor suppressive effect of FGF14 was correlated to the inducement of apoptosis, as proved in FGF14-expressing CRC cell lines and xenografted tumors in the nude mice. Caspase activation is considered a cardinal feature of apoptosis, including upregulation of the active forms of casepase-3, casepase-7 and poly ADP ribose polymerase (PARP) 22. Here, we found that pro-apoptotic markers, cleaved casepase-3, casepase-7, PARP and Bax were upregulated with FGF14 overexpression, while anti-apoptotic marker Bcl-2 decreased significantly. It is well-recognized that Caspase-3, caspases-7 and PARP are responsible for cell collapse and death 23. These data supported that FGF14 elicits apoptosis through a caspase-dependent mitochondrial manner.

Given the critical role of PI3K/AKT signaling pathway in regulating cell survival/death 24, we further investigated whether FGF14 interacts with the PI3K/AKT signaling pathway to induce cell apoptosis of CRC. We found that key molecules of PI3K/AKT/mTOR signaling pathway (including PI3K, phosphorylated AKT and phosphorylated mTOR) were downregulated with ectopic expression of FGF14 in both CRC cell lines. These results indicated that FGF14 might induce mitochondrial apoptosis through inhibiting PI3K/AKT/mTOR signaling pathway in CRC. Of note, negative relationship between p-PI3K and p-Akt was observed in CRC cells with ectopic expression of FGF14 (Fig. 3C), which seems contradicted with the traditional opinion that AKT is a major downstream effector of PI3K. Some studies, however, have illustrated an independent role between PI3K and AKT in tumors. For example, in breast cancers, it has been reported that no correlation was found between AKT phosphorylation and the activation of PI3K mutations. However, phosphorylated AKT was found significantly higher in tumors with low level of PTEN 25. Accordingly, it was demonstrated that AKT phosphorylation could be strikingly downregulated in tumor cells harboring PI3K activating mutations 26. Therefore, the negative relationship between phosphorylated PI3K and AKT might be attributed to the existence of mutated PI3K or the regulation of PTEN, which warrants further investigation.

In conclusion, the data reported here provided strong support to the concept that reduced or silenced expression of FGF14 is modulated by DNA methylation of this gene, and FGF14 functions as a novel tumor suppressor, which induces cell apoptosis *via* inhibiting PI3K/AKT/mTOR signaling. To our best knowledge, this study represents the first comprehensive analysis of epigenetic regulation, biological function and molecular mechanism of FGF14 in CRC, which may shed light on the understanding of FGF14 and PI3K/AKT/mTOR signaling pathway in colorectal carcinogenesis.

## Supplementary Material

Supplementary figures and tables.

## Abbreviations

FGF14: Fibroblast Growth Factor 14
CRC: colorectal cancer
FGF: fibroblast growth factor
FAHSYSU: the First Affiliated Hospital, Sun Yat-Sen University
MEF: mouse embryonic fibroblasts
STR: short tandem repeat
5-Aza: 5-Aza-2'-deoxycytidine
CCK-8: cell counting Kit-8
PARP: poly (ADP-ribose) polymerase
