# Supplementary material for: FGF14 Functions as a Tumor Suppressor through Inhibiting PI3K/AKT/mTOR Pathway in Colorectal Cancer
Source: J Cancer. 2020 Jan 1;11(4):819–25. doi: 10.7150/jca.36316 (PMC6959027; doi:10.7150/jca.36316)
Supplement: Supplementary file 1 — Supplementary figures and tables. [file jcav11p0819s1.pdf]

Fig. S1. Methylation-specific PCR (MSP) was performed to determine promoter methylation status of FGF14. M means the methylated DNA; U means the unmethylated DNA (left panel). Treatment with methyltransferase inhibitor 5-Aza-20-deoxycytidine (5-Aza) restored the mRNA expression of FGF14 (right panel). GAPDH was used as loading control.

Fig. S2. FGF14 influenced little on the growth of MEF cells. (A) The silence effect of small interference RNA on FGF14 in MEF cells was detected by Western blot. (B) Silencing FGF14 showed little impact on the proliferation of MEF cells. (C) Silencing FGF14 had little influence on the colony formation ability of MEF cells.

Fig. S3. No correlation was observed between the ectopic expression of FGF14 and cell migration or cell invasion of CRC. (A) Migration ability of CRC cells was not affected by the ectopic expression of FGF14, as shown by migration assay in DLD1 and HCT116 cells. (B) Invasion ability of CRC cells was not affected by the ectopic expression of FGF14, as shown by invasion assay in DLD1 and HCT116 cells.

Fig. S1

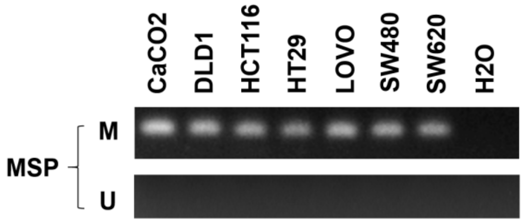

Fig. S2

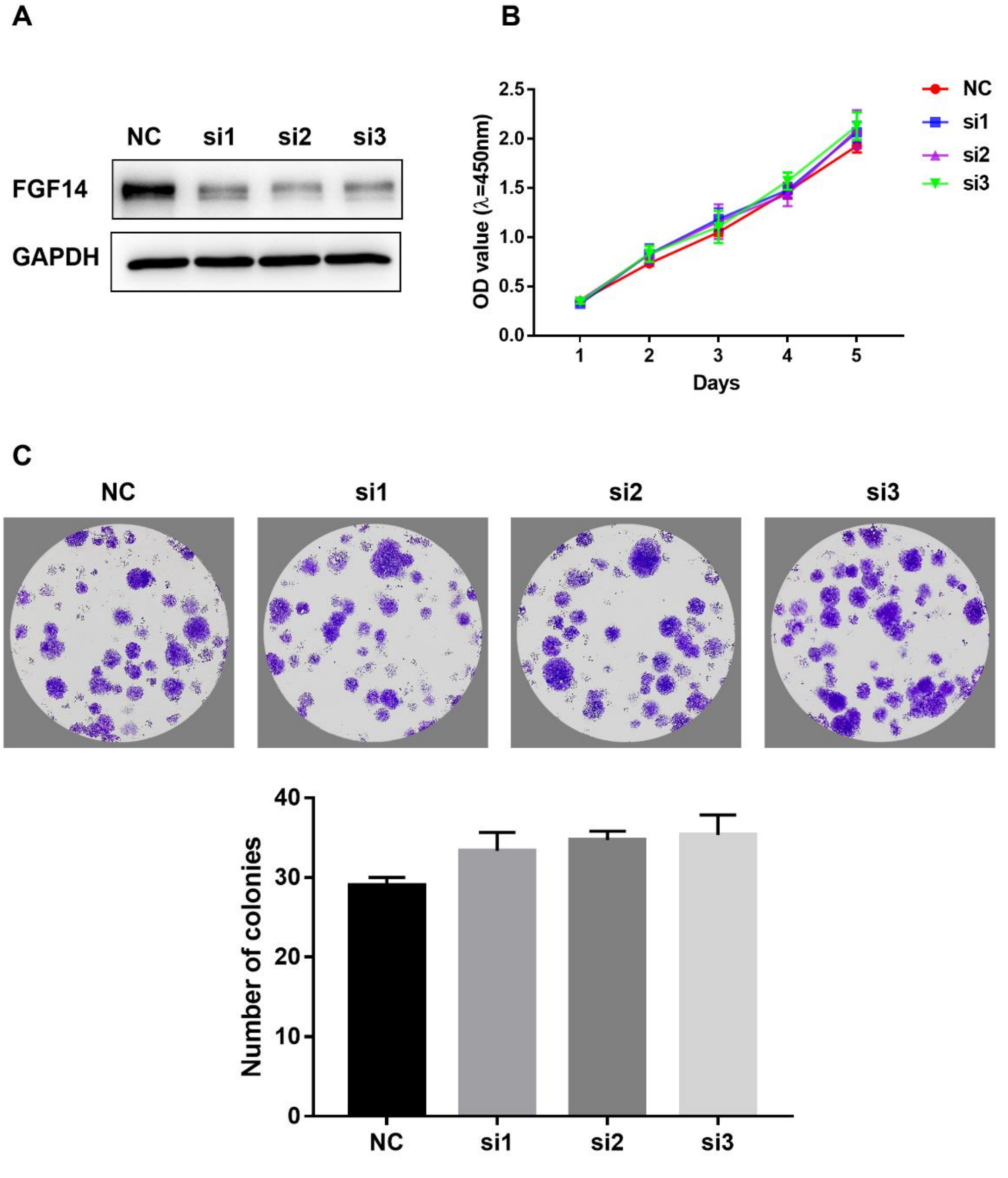
A
NC si1 si2 si3
FGF14
GAPDH
B
OD value (λ=450nm)
2.5
2.0
1.5
1.0
0.5
0.0
1 2 3 4 5
Days
NC
si1
si2
si3
C
NC si1 si2 si3
Number of colonies
40
30
20
10
0
NC si1 si2 si3

Fig. S3

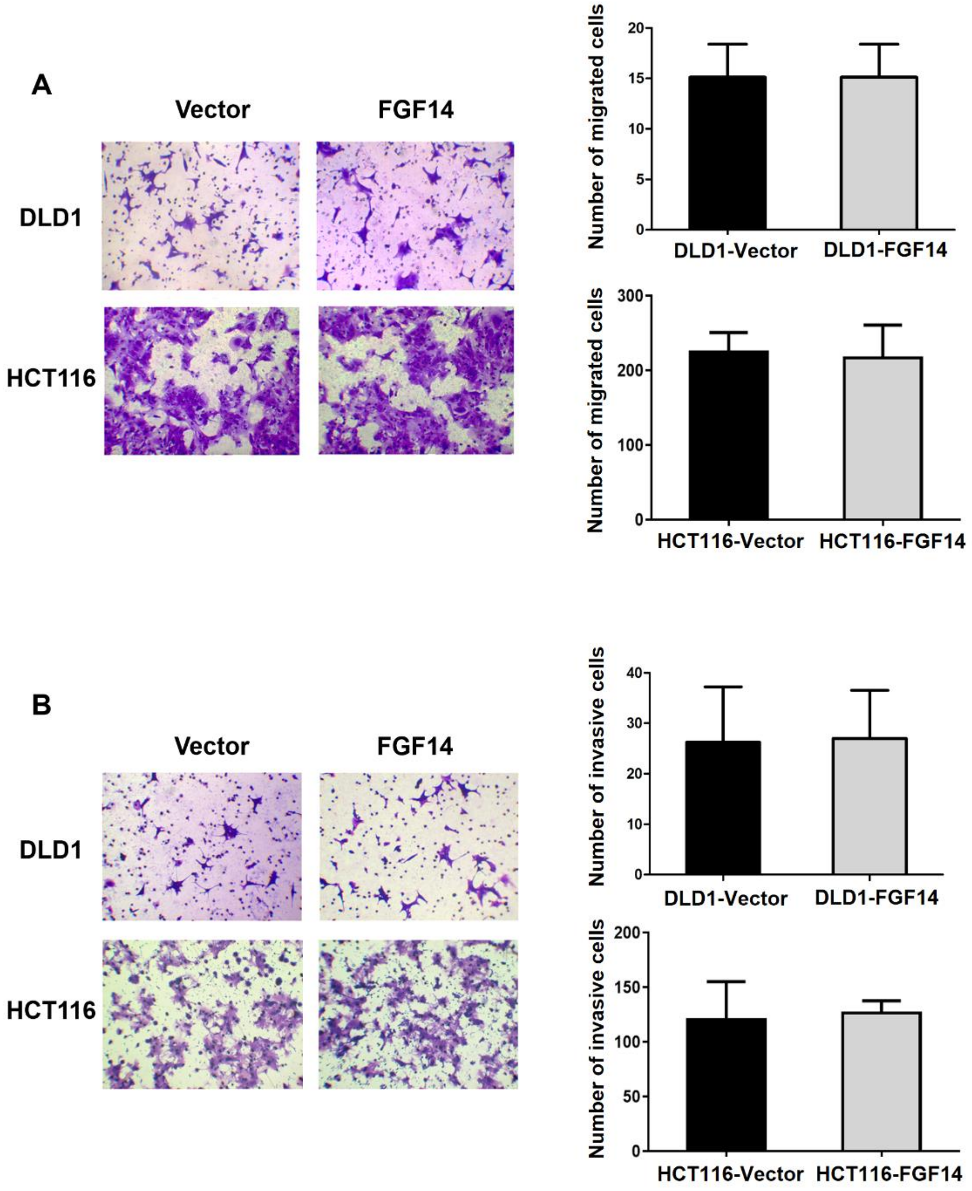

A
Vector
FGF14
DLD1
HCT116
Number of migrated cells
20
15
10
5
0
DLD1-Vector
DLD1-FGF14
300
200
100
0
HCT116-Vector
HCT116-FGF14
B
Vector
FGF14
DLD1
HCT116
Number of invasive cells
40
30
20
10
0
DLD1-Vector
DLD1-FGF14
200
150
100
50
0
HCT116-Vector
HCT116-FGF14

Supplementary Table 1. Primers Used

FGF14-F	GGCAACCTGGTGGATATCTT
FGF14-R	CCTTGCCTGCAATATAACCT
GAPDH-F	CAGCCTCAAGATCATCAGCA
GAPDH-R	TGTGGTCATGAGTCCTTCCA
FGF14-MSP-F	GTATCGGTAATTACGTTAAGATCGA
FGF14-MSP-R	ATAAAATTAAAAAAACATCGAAACG
FGF14-USP-F	GTATTGGTAATTATGTTAAGATTGA
FGF14-USP-R	AAAATTAAAAAAACATCAAAACACT

Supplementary Table 2. Antibodies Used

Antibody	Code	Source information
FGF14	ab229610	Abcam
GAPDH	#8884	Cell Signaling Technology
mTOR	#2972	Cell Signaling Technology
p-mTOR	#2971	Cell Signaling Technology
Akt	#9272	Cell Signaling Technology
p-Akt	#4060	Cell Signaling Technology
PI3K	#4255	Cell Signaling Technology
p-PI3K	#4249	Cell Signaling Technology
Bax	#5023	Cell Signaling Technology
Bcl-2	#4223	Cell Signaling Technology
cleaved PARP	#5625	Cell Signaling Technology
cleaved Caspase-3	#9664	Cell Signaling Technology
cleaved Caspase-7	#9497	Cell Signaling Technology
